# Initial evaluation of [^18^F]-FACBC for PET imaging of multiple myeloma

**DOI:** 10.1186/s13550-022-00876-0

**Published:** 2022-01-31

**Authors:** Volker Morath, Michael Heider, Markus Mittelhäuser, Hannes Rolbieski, Jacob Stroh, Jérémie Calais, Matthias Eiber, Florian Bassermann, Wolfgang A. Weber

**Affiliations:** 1 Department of Nuclear Medicine, Klinikum rechts der Isar, School of Medicine, Technical University of Munich, 81675 Munich, Germany; 2 Department of Medicine III, Klinikum rechts der Isar, School of Medicine, Technical University of Munich, 81675 Munich, Germany; 3 Ahmanson Translational Theranostics Division, Department of Molecular and Medical Pharmacology, University of California Los Angeles, Los Angeles, CA USA; 4 TranslaTUM, Center for Translational Cancer Research, Technical University of Munich, 81675 Munich, Germany

**Keywords:** FACBC, Fluciclovine, Multiple myeloma, Hematology, FET, PET

## Abstract

**Rationale:**

Multiple myeloma (MM) cells synthesize large amounts of paraproteins, making radiolabeled amino acids promising candidates for PET imaging of MM patients.

**Methods:**

We compare tumor uptake of the two amino acid analogs [^18^F]-fluoroethyltyrosine and [^18^F]-FACBC in a MM xenograft model and show the feasibility of PET imaging with [^18^F]-FACBC in a MM patient.

**Results:**

Preclinically [^18^F]-FACBC showed superior performance, mainly due to the uptake via the ASC-system. In a subsequent proof-of-concept investigation [^18^F]-FACBC PET was performed in a MM patient. It allowed identification of both lesions with and without CT correlate (SUVmean 8.0 or 7.9) based on higher uptake compared to normal bone marrow (SUVmean 5.7). Bone signal was elevated compared to non-MM patients, and, thus [^18^F]-FACBC potentially allows the assessment of bone marrow infiltration.

**Conclusion:**

The FDA/EMA approved PET agent [^18^F]-FACBC is promising for imaging MM and should be further evaluated in prospective clinical studies.

## Introduction

Multiple Myeloma (MM) is a hematological neoplasm of clonally proliferating plasma cells with focal or diffuse localization in the bone marrow. Following their plasma cell phenotype approximately 98% of MMs retain their secretory activity meaning that they produce and secrete large amounts of immunoglobulin-like proteins, so-called paraproteins [1]. Amino acids (AAs) needed for paraprotein biosynthesis add to the AA required for cell proliferation, as in other malignancies. Consequently, AA uptake of MM is exceptionally high and a hallmark of MM biology [1, 2].

Imaging plays an important role for diagnosis, staging and restaging of MM [1]. Osteolytic lesions on computer tomography (CT) or magnetic resonance imaging (MRI) are a defining feature of MM [1]. FDG PET/CT can detect viable, metabolically active MM and has demonstrated encouraging results for monitoring MM therapy [3]. However, FDG PET/CT has limitations for the detection of diffuse bone marrow and meningeal involvement and can be false-positive in patients with compression fractures, a frequent complication of MM [4]. Therefore, a variety of other PET-radiopharmaceuticals such as [^11^C]-methionine, [^11^C/^18^F]-choline, and [^68^ Ga]-pentixafor have been clinically evaluated for MM imaging [4]. In a comparative study [^11^C]-methionine PET/CT has shown a higher sensitivity for intra- and extramedullary MM than FDG PET/CT [5]. However, broader clinical use of [^11^C]-methionine is severely limited by its short, 20 min physical half-life. For the AA analogue [^18^F]-fluorethlytyrosine (FET) conflicting preclinical data about its uptake in MM cells have been reported [6, 7]. However, a recent clinical study reported high uptake of FET by MM and excellent image contrast [8].

[^18^F]-FACBC (Anti-1-amino-3-[^18^F]-fluorocyclobutane-1-carboxylic acid) is a leucine analogue which is FDA and EMA approved for imaging of recurrent prostate cancer [9, 10]. Leucine is an essential AA and has the highest abundance in the human proteome (~ 10%), as compared to about 2% for methionine [11]. While [^18^F]-FET and [^18^F]-methionine are mainly transported by the sodium-independent L-system, [^18^F]-FACBC is equally transported by the sodium-independent L-system and the sodium-dependent ASC-system [12, 13]. Both of these AA transport systems are linked to aggressive tumoral behavior and have been associated with decreased survival rates [14]. The simultaneous import of [^18^F]-FACBC by both transport systems promises a rapid and mechanistically robust uptake into tumors with up-regulated AA transport systems.

Despite these promising characteristics, FACBC has to our knowledge not been thoroughly investigated for imaging of MM so far. Therefore, we compared tumor uptake and biodistribution with [^18^F]-FET in a MM xenograft model and performed a proof-of-concept investigation assessing the feasibility and clinical potential of PET imaging with [^18^F]-FACBC in a patient with MM.

## Materials and methods

### Radiotracers

[^18^F]-FACBC (Axumin) was bought from AAA (Advanced Accelerator Applications, Saint-Genis-Pouilly, France). The radioligand was available from the AAA site in Seibersdorf (near Vienna, Austria) and was transported directly to Munich. [^18^F]-FET (O-(2-[^18^F]-fluoroethyl)-l-tyrosine) was produced in house as previously described [15]. Reformulation of both radiopharmaceuticals into phosphate-buffered saline was carried out by ion exchange cartridges to remove the citrate buffer which is known for its toxicity for rodents.

### Animal studies

Variants of the human MM cell line MM.1S have been generated and described before [7]. In short, the cell line MM.1S^LAT1/CD98hc^ overexpressing the two components of the heterodimeric AA transporter of the L-system was used together with a control MM.1S cell line expressing empty vectors, MM.1S^ev/ev^. Female NOD-SCID mice (Charles River Laboratories, Wilmington, MA) were subcutaneously injected with 6 × 10^6^ MM.1S^ev/ev^ and MM1S^LAT1/CD98hc^ cells mixed 1:1 with Matrigel hc (Corning, NY) above the right and left shoulder, respectively. After 3 weeks mice were equally divided into two cohorts of N = 8 and PET data was recorded using an Inveon PET/CT small-animal scanner (Siemens Medical Solutions, Erlangen, Germany). FACBC was injected (4.5 ± 0.26 MBq) i.v. and a first animal was scanned dynamically from 0–50 min. Subsequent FACBC scans were started 30 min p.i. and the signal was recorded for 10 min, as a rapid washout of FACBC was described in literature. FET was injected (12 ± 0.42 MBq) i.v. and after 45 min the signal was recorded for 15 min (institutional standard protocol for FET scans).

Image reconstruction was performed using a 0.8 mm high resolution OSEM-3D algorithm. Volumes of interest (VOI) for the xenograft tumors were segmented with 3D isocontours set at 50% of the maximum. Statistical analysis was performed by unpaired t-tests using GraphPad Prism.

## Results

### Preclinical comparison of [^18^F]-FACBC versus [^18^F]-FET

**Fig. 1:**
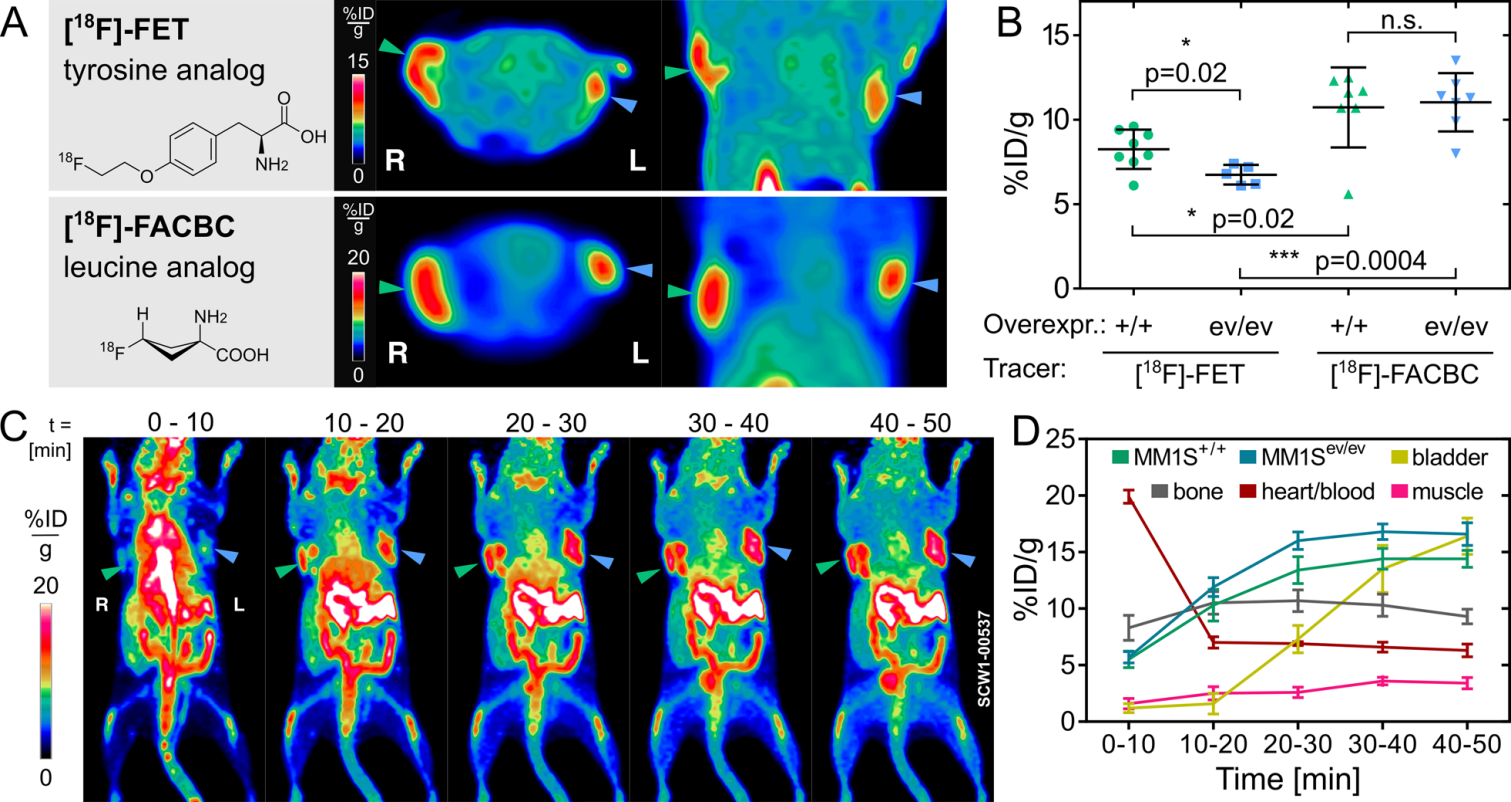
Preclinical comparison of [^18^F]-FET versus [^18^F]-FACBC in MM. **A** Structure of the radiotracers [^18^F]-FET and [^18^F]-FACBC together with a representative axial section and a maximum intensity projection (MIP) of the shoulder region. For both radiotracers, eight animals were injected with MM.1S cells transduced with control (ev/ev, blue arrowheads, left shoulder) and LAT1/CD98hc transduced (MM.1S^LAT1/CD98hc^, green arrowheads, right shoulder). **B** From 8 mice injected per cohort, 5–8 xenograft tumors had grown, which were imaged and segmented. Student’s t-test with *p < 0.05; **p < 0.01; ***p < 0.005, error bars indicate SD. **C** Dynamic [^18^F]-FACBC PET-scan of a mouse with 10 min frames over 50 min post injection represented as a MIP. **D** [^18^F]-FACBC time-activity curve for different tissues measured by a representative sphere with 5 px diameter, error bars indicate SD

Both AA radiopharmaceuticals allowed the visualization of the xenografts with high contrast (Fig. 1A). [^18^F]-FACBC yielded a significantly higher quantitative uptake into both types of MM.1S tumors (10.7 ± 0.9 and 11.0 ± 0.7%ID/g; mean ± SEM) compared to the [^18^F]-FET cohort (8.3 ± 0.4 and 6.7 ± 0.3%ID/g). This difference in uptake between [^18^F]-FACBC and [^18^F]-FET was highly significant for the MM.1S^ev/ev^ xenograft tumors (*p* = 0.0004). These cells showed a 64% higher uptake of [^18^F]-FACBC over [^18^F]-FET (11.0 vs. 6.7%ID/g) (Fig. 1B). Interestingly, uptake of [^18^F]-FACBC was not further increased by overexpression of the LAT-system in the MM.1S^CD98hc/LAT1^ tumors. In contrast, transduction with CD98hc/LAT1 significantly increased tumor uptake of [^18^F]-FET (Fig. 1B). This suggests that the second transport system for [^18^F]-FACBC, the ASC-system, plays an important role for [^18^F]-FACBC uptake in MM and may be responsible for the generally higher uptake of [^18^F]-FACBC.

In a dynamic PET-scan with [^18^F]-FACBC the signal within the tumor increased to app. 30 min and remained constant until the end of the scan at *t* = 50 min after injection (Fig. 1C). Tumor-to-muscle ratios (16.8 ± 0.7 or 14.4 ± 0.9 vs. 3.6 ± 0.3%ID/g, mean ± SD) were 4.7 or 4.0 and tumor-to-blood ratios (16.8 ± 0.7 or 14.4 ± 0.9 vs. 6.6 ± 0.4%ID/g) 2.5 or 2.2 for the MM1S^LAT1/CD98hc^ or the MM1S^ev/ev^ xenograft, respectively, as determined from the dynamic PET-scan at the 30–40 min frame.

### Proof-of-concept [^18^F]-FACBC PET-imaging in a MM patient

Based on the promising preclinical results we performed a proof-of-concept investigation assessing the clinical potential of [^18^F]-FACBC PET in a 54-year old male patient with newly diagnosed MM, several osteolytic lesions of the thoracic and lumbar spine as well as diffuse bone marrow involvement (15–20%) on iliac crest biopsy. Immunohistochemistry revealed the presence of MM cells with positivity for CD138, MUM1, CyclinD1 and light chain lambda restriction. Serum and urine immunofixation detected a monoclonal IgG lambda gammopathy and quantitative plasma proteins were elevated (IgG 5069 mg/dl, light chain lambda 607 mg/l). Apart from osteolytic bone lesions, no CRAB criteria were met. The PET/CT scan was performed to better define the extent of disease prior to therapy.

The patient underwent a [^18^F]-FACBC PET/CT on a Biograph mCT scanner (Siemens Medical Solutions, Erlangen, Germany). The PET scan was started 3 min after injection of 371 MBq [^18^F]-FACBC and a whole-body scan with 3 min per bed position was acquired. Lesion uptake was quantified using a 3D VOI with 50% isocontours.

**Fig. 2:**
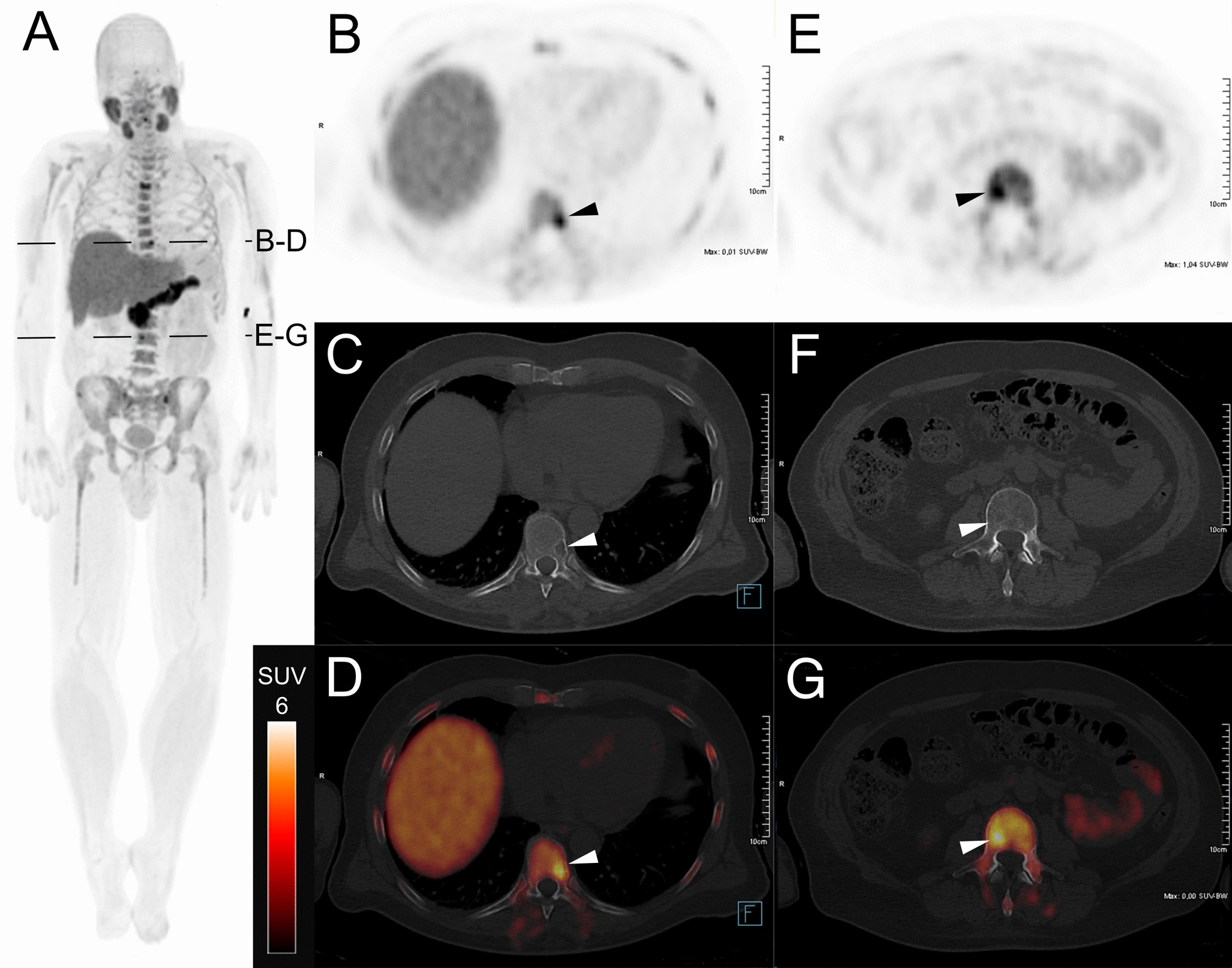
[^18^F]-FACBC PET/CT-scan of a MM patient. **A** Full body MIP. Axial sections of this patient’s PET (**B**/**E**), CT (**C**/**F**) and PET/CT fusion (**D**/**G**) are depicted. Position of the axial sections is indicated in the MIP (**A**). Concordant lesion on PET and CT (**B**–**D**; SUVmean 8.0; SUVmax 13.1), where the PET-signal corresponds to osteolysis on CT. [^18^F]-FACBC allowed the localization of discordant lesions in PET that were not distinguishable from bone inhomogeneity in CT (**E**–**F**; SUVmean 7.9; SUVmax 9.5)

The PET scan revealed diffusely increased uptake of [^18^F]-FACBC in the bone marrow (SUVmean 5.7; SUVmax 9.0) which was more than two standard deviations higher than reported for the normal bone marrow (L3 vertebrae; SUVmean 3.9 ± 0.8 (SD) [16],). In addition, several distinct focal lesions were found (Fig. 2). A lesion with osteolysis correlated with CT showed a SUVmean 8.0 and a SUVmax 13.1 (Fig. 2B–D). Furthermore, the PET scan detected foci without clear CT-morphologic osteolytic lesions showing a discordant lesion which exhibited a comparable [^18^F]-FACBC uptake (SUVmean 7.9, SUVmax 9.5) as the osteolytic lesions on CT (Fig. 2E–G).

## Discussion

This is the first comparative study evaluating the AA analogue [^18^F]-FACBC for imaging of multiple myeloma (MM). While preliminary, our results clearly suggest that [^18^F]-FACBC allows for high-contrast imaging of MM lesions and warrant further clinical studies of [^18^F]-FACBC in MM patients. Although limited to an initial patient due to the availability of [^18^F]-FACBC, this study may prompt a clinical trial that systemically assesses the use of [^18^F]-FACBC in MM. Potential applications to be tested in this clinical trials include differentiation of monoclonal gammopathy of unknown significance (MGUS) and smoldering myeloma from active MM, assessment of the extent of disease in patients with known MM, monitoring the therapeutic response and detection of minimal residual disease (MRD).

While FDG PET has shown clinical utility for these applications [3], there are also well-known limitations, such as false negative findings due to low FDG uptake of some myeloma types as well as false positive findings due to inflammation and healing compression fractures. Because MM can involve the whole skeleton and may present with small solitary lesions in peripheral bones, there is a clear need for a radiotracer that provides high-contrast whole-body images of MM while showing lower uptake than FDG in benign changes.

[^18^F]-FACBC is an FDA/EMA-approved drug for prostate cancer and could therefore easily be repurposed for MM imaging. Furthermore, there is an established distribution network for [^18^F]-FACBC which could make this radiotracer widely available for MM patients. This is in marked contrast to [^11^C]-methionine which is only available at PET centers with an on-site cyclotron. While in-vitro studies have shown markedly higher uptake rates of [^11^C]-methionine as compared to fluorine-18 labeled amino acids in MM [6] and lymphoma cells [17] in-vivo uptake of [^18^F]-FET was higher than of [^11^C]-methionine in a murine lymphoma model [18]. Clinical studies of glioma patients have shown very similar tumor uptake of [^11^C]-methionine and [^18^F]-FET [19, 20]. Future clinical studies are required to determine if the diagnostic performance of [^18^F]-FACBC is comparable to [^11^C]-methionine in MM. Similarly, future studies are required to compare the diagnostic performance of FACBC in MM with MR imaging and FDG PET. These prospective and ideally head-to-head studies of [^18^F]-FACBC in comparison to other radiotracers are required to determine the sensitivity and specificity of [^18^F]-FACBC PET for detection of MM lesions and to establish quantitative thresholds of tracer uptake for the diagnosis of diffuse bone marrow involvement.

An alternative AA tracer for MM imaging is [^18^F]-FET which also showed high tumor uptake in our preclinical studies as well as in a recent clinical trial [8]. Tumor uptake of [^18^F]-FET was, however, significantly lower than of [^18^F]-FACBC in our preclinical study. [^18^F]-FET which is used at several European centers for brain tumor imaging is not FDA/EMA-approved and accordingly no distribution network exists.

Whole-body low-dose CT and MRI are clinically relevant imaging modalities that have replaced planar x-ray studies in MM. Whole-body CT is robust and can be acquired quickly but it is challenging to reliably detect small osteolytic lesions in the whole skeleton. The sensitivity of CT for extramedullary MM is low. MRI provides excellent sensitivity for detection of MM in the axial skeleton, but sensitivity is limited for rib lesions. Furthermore, whole body MRI including the peripheral skeleton is time consuming because several MR sequences need to be acquired and evaluated. Thus, there remains an unmet clinical need for new PET radiopharmaceuticals for MM.

## Conclusion

We show that [^18^F]-FACBC is a promising candidate for PET imaging of MM that should be further evaluated in prospective clinical trials.

## Data Availability

The datasets used and/or analyzed during the current study are available from the corresponding author on reasonable request.

## Abbreviations

AA: Amino acid
AAA: Advanced Accelerator Applications
CT: Computer tomography
EMA: European Medicines Agency
FACBC: (Anti-1-amino-3-[^18^F]-fluorocyclobutane-1-carboxylic acid)
FDA: Food and Drug Administration
FDG: [^18^F]-Fluorodeoxyglucose (2-deoxy-2-[^18^F]fluoro-d-glucose)
FET: [^18^F]-fluorethlytyrosine (O-(2-[^18^F]-fluoroethyl)-l-tyrosine)
MGUS: Monoclonal gammopathy of unknown significance
MM: Multiple myeloma
MRD: Minimal residual disease
MRI: Magnetic resonance imaging
PET: Positron emission tomography
SUV: Standardized uptake value
VOI: Volumes of interest
